# Malaria incidence in Myanmar 2005–2014: steady but fragile progress towards elimination

**DOI:** 10.1186/s12936-016-1567-0

**Published:** 2016-10-18

**Authors:** Thet Thet Mu, Aye Aye Sein, Tint Tint Kyi, Myo Min, Ne Myo Aung, Nicholas M. Anstey, Myat Phone Kyaw, Chit Soe, Mar Mar Kyi, Josh Hanson

**Affiliations:** 1 Department of Public Health, Ministry of Health, Nay Pyi Taw, Myanmar; 2 Department of Medical Care, Ministry of Health, Nay Pyi Taw, Myanmar; 3 Myanmar Medical Association, Yangon, Myanmar; 4 University of Medicine 2, Yangon, Myanmar; 5 Menzies School of Health Research, Darwin, Australia; 6 Department of Medical Research, Yangon, Myanmar; 7 University of Medicine 1, Yangon, Myanmar; 8 The Kirby Institute, Sydney, Australia

**Keywords:** Malaria, Myanmar, Artemisinin resistance, Epidemiology, Public health, Greater Mekong Region

## Abstract

**Background:**

There has been an impressive recent reduction in the global incidence of malaria, but the development of artemisinin resistance in the Greater Mekong Region threatens this progress. Increasing artemisinin resistance is particularly important in Myanmar, as it is the country in the Greater Mekong Region with the greatest malaria burden. If malaria is to be eliminated in the region, it is essential to define the spatial and temporal epidemiology of the disease in Myanmar to inform control strategies optimally.

**Results:**

Between the years 2005 and 2014 there was an 81.1 % decline in the reported annual incidence of malaria in Myanmar (1341.8 cases per 100,000 population to 253.3 cases per 100,000 population). In the same period, there was a 93.5 % decline in reported annual mortality from malaria (3.79 deaths per 100,000 population to 0.25 deaths per 100,000 population) and a 87.2 % decline in the proportion of hospitalizations due to malaria (7.8 to 1.0 %). Chin State had the highest reported malaria incidence and mortality at the end of the study period, although socio-economic and geographical factors appear a more likely explanation for this finding than artemisinin resistance. The reduced malaria burden coincided with significant upscaling of disease control measures by the national government with support from international partners. These programmes included the training and deployment of over 40,000 community health care workers, the coverage of over 60 % of the at-risk population with insecticide-treated bed nets and significant efforts to improve access to artemesinin-based combination treatment. Beyond these malaria-specific programmes, increased general investment in the health sector, changing population demographics and deforestation are also likely to have contributed to the decline in malaria incidence seen over this time.

**Conclusions:**

There has been a dramatic fall in the burden of malaria in Myanmar since 2005. However, with the rise of artemisinin resistance, continued political, financial and scientific commitment is required if the ambitious goal of malaria elimination in the country is to be realized.

## Background

There has been significant recent progress in the fight against malaria [1]. In Southeast Asia the annual number of malaria cases declined by almost 50 % between 2000 and 2014 [1], and as a result there is now a plan in the Greater Mekong Region for elimination of *Plasmodium falciparum* by 2025 and for elimination of all malaria by 2030 [2]. However, while there have been impressive gains, major challenges remain and if the momentum of the last 15 years is to be maintained, sustained global, regional and local commitment is required. The Greater Mekong Region has many poor, vulnerable and geographically remote populations and it is these people who bear the greatest burden of disease [1, 3, 4]. The region also has the unique challenge of artemisinin resistance that threatens not only recent local gains, but which also has the potential to reverse positive global trends if it spreads to the rest of the world [5–7].

Myanmar has the greatest malaria incidence in the Greater Mekong Region [1, 8] and its new government and poorly resourced public health system will have to overcome a variety of political, economic and logistic challenges if malaria is to be eliminated [9]. Several interventions have already been implemented and these include the training and deployment of community health workers [10, 11], the provision of insecticide-treated bed nets [12] and strategies to improve access to rapid diagnostic tests [13] and artemisinin-based combination therapy (ACT) [14]. There has also been a concerted effort to improve diagnosis and management of the disease in the private sector where the majority of malaria cases are managed [15]; here there has been a particular focus on the removal of poor quality ACT and artesunate monotherapy [14, 16]. While mathematical modelling has a valuable role to play in determining the efficacy of such interventions to decrease the malaria burden [17, 18], these models require detailed and reliable data so that decision-makers may target disease control programmes optimally.

The Health Management Information System (HMIS) section of the Department of Health in Myanmar produces a monthly report based on data collected in the field by health care workers in the country’s public health system. To document the changing epidemiology of clinical malaria in Myanmar, the last ten years of available HMIS malaria data (2005–2014) were analysed to demonstrate both the progress that has been made and the challenges that remain.

## Methods

Data from between 1 January, 2005 and 31 December, 2014 were collected from the HMIS database, which documents every reported case of malaria in the country’s public health system. Each case is notified to the local rural health centre that generates a report that is sent to the township health department. A township level report is then forwarded centrally to the HMIS and to the health department of each of Myanmar’s states and regions. From 2012, only patients with a diagnosis of malaria confirmed by microscopy or rapid diagnostic test were included as cases. Prior to 2012, limited access to definitive diagnostic testing in many areas of the country meant that ‘probable cases’ (based on clinical presentation and disease course) were also included as cases. The HMIS database does not differentiate malaria cases by species. Hospital inpatient data are also collected monthly from all hospitals and this is forwarded to the HMIS; these data capture the principal diagnosis of every hospital admission in the country and includes the cause of all deaths.

Incidence calculations were based on official contemporaneous estimations of population size. No census was performed in Myanmar between 1982 and 2014 and so these official population data were based on the 1982 census and official projected estimates of fertility, migration and mortality [19]. Data were collected in Microsoft Excel and Figures were constructed with Microsoft Excel and Map Window.

### Ethics

The Chair of the Menzies School of Health Research Human Research Ethics Committee (HREC) deemed that the work could be exempted from the review of the full HREC as it posed negligible risk to participants.

## Results

### Disease incidence

**Fig. 1:**
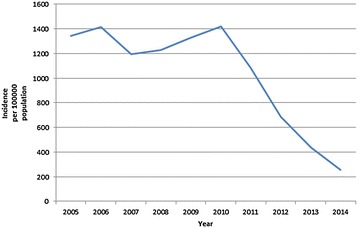
National incidence (per 100,000 population) of malaria in Myanmar 2005–2014

**Fig. 2:**
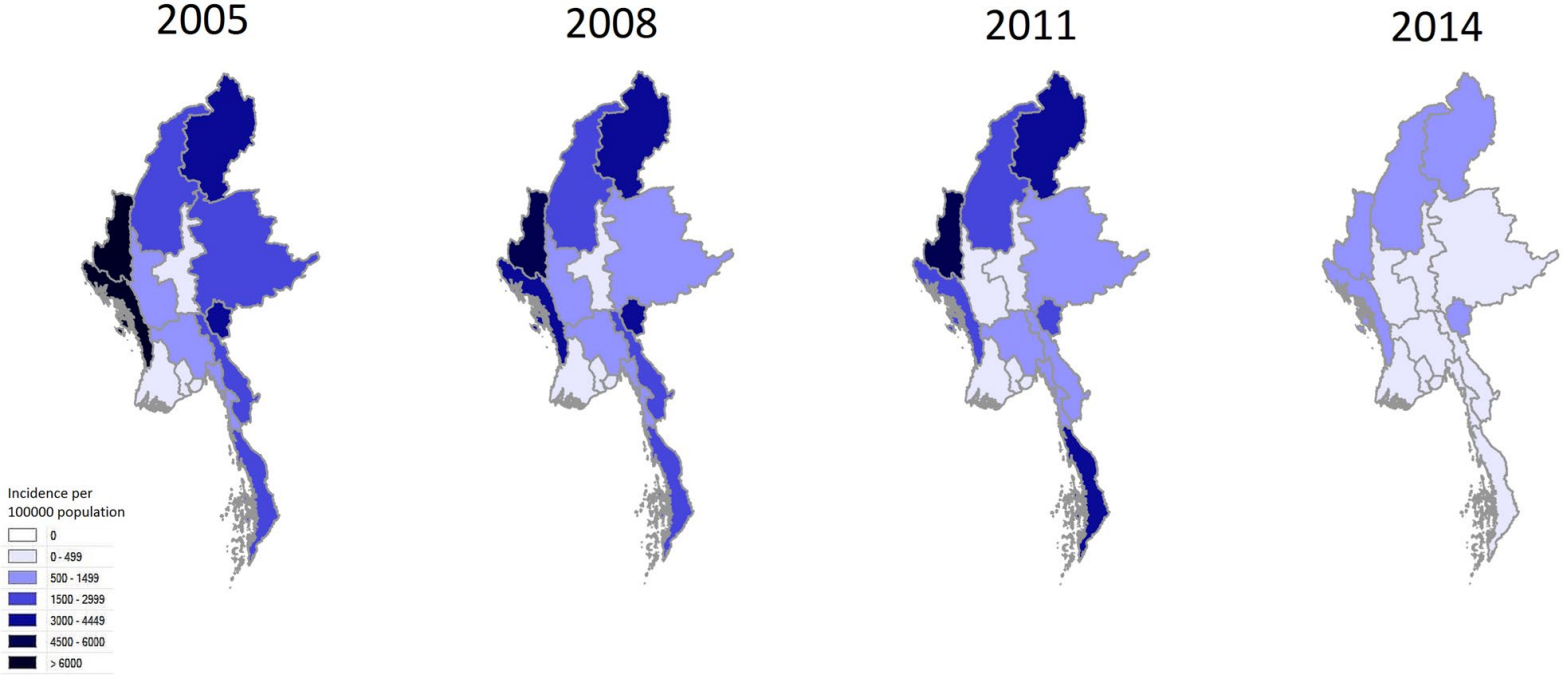
Geographical distribution of malaria cases 2005–2014 (expressed as incidence per 100,000 population)

Over the course of the study period, the reported national malaria incidence fell from 1341.8 cases per 100,000 population, to 253.3 cases per 100,000 population, a decline of 81.1 % (Fig. 1). The reported incidence fell in all of the states and regions of the country, ranging from a 61.8 % decline in the Ayeyarwaddy Region to a 94.4 % decline in Mon State (Fig. 2; Table 1).

**Table 1:** Malaria incidence (per 100,000 population) by region/state 2005–2014

Region/state	2005	2006	2007	2008	2009	2010	2011	2012	2013	2014	% decline^a^
Ayeyarwaddy	437.8	382.1	436.6	352.6	301.1	483.1	585.5	361.3	298.3	167.4	61.8
Bago	854.9	821.9	704.9	1178.8	1108.3	980.6	784.6	484.5	246.6	68.6	92.0
Chin	6109.8	5499.2	5072.6	4811.0	5467.0	4615.4	4584.2	2613.6	1583.7	1499.6	75.5
Kachin	4375.4	4968.6	3806.2	3899.5	6259.0	7335.6	3804.9	2374.2	1691.6	832.2	81.0
Kayah	3197.0	3667.4	3504.0	3098.4	3272.8	3057.3	2799.6	1858.6	1068.2	517.1	83.8
Kayin	1702.7	1827.1	1484.4	1544.2	1562.4	1716.4	1472.9	970.0	869.4	435.2	74.4
Magway	738.9	650.3	758.6	808.6	873.1	1010.3	526.7	322.1	161.8	85.9	88.4
Mandalay	337.3	263.7	288.9	310.4	353.2	372.1	372.6	279.5	147.7	74.4	78.0
Mon	1147.6	1049.4	1172.7	1139.2	1161.6	1245.4	905.1	362.2	175.7	64.2	94.4
Nay Pyi Taw^b^	n/a	n/a	n/a	n/a	n/a	n/a	n/a	250.3	147.8	91.7	n/a
Rakhine	6511.3	7811.4	5141.1	4135.5	3736.0	3727.7	2846.9	1752.8	877.8	628.1	90.4
Sagaing	1552.2	1383.7	1324.3	1564.9	1962.4	2313.3	1523.6	1085.6	755.1	536.0	65.5
Shan	1629.4	1619.3	1376.9	1474.2	1452.2	1421.4	1095.5	799.9	588.7	365.2	77.6
Tanintharyi	2172.0	2713.0	2263.5	2391.9	2504.0	2888.6	3581.6	2166.9	1291.4	421.4	80.6
Yangon	122.7	124.7	96.3	104.8	91.1	76.4	38.3	25.4	12.0	8.6	93.0
*National*	*1341.8*	*1415.4*	*1192.9*	*1226.5*	*1327.3*	*1420.0*	*1085.2*	*686.0*	*438.3*	*253.3*	*81.1*

^a^Between 2005 and 2014

^b^Nay Pyi Taw was formed as an administrative region in 2010; until 2012 data were included in the Mandalay region

### Malaria mortality

**Fig. 3:**
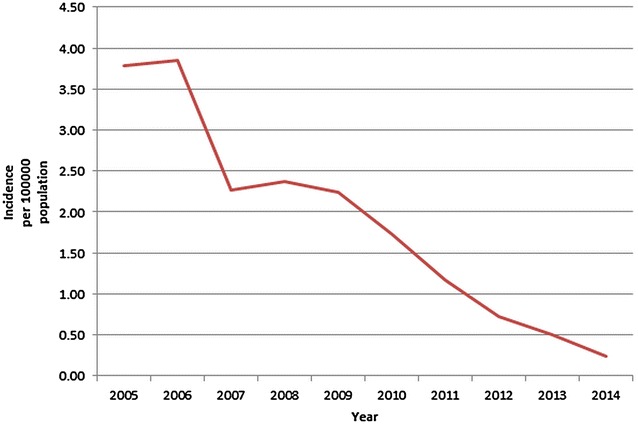
National mortality rate (per 100,000 population) of malaria in Myanmar 2005–2014

**Fig. 4:**
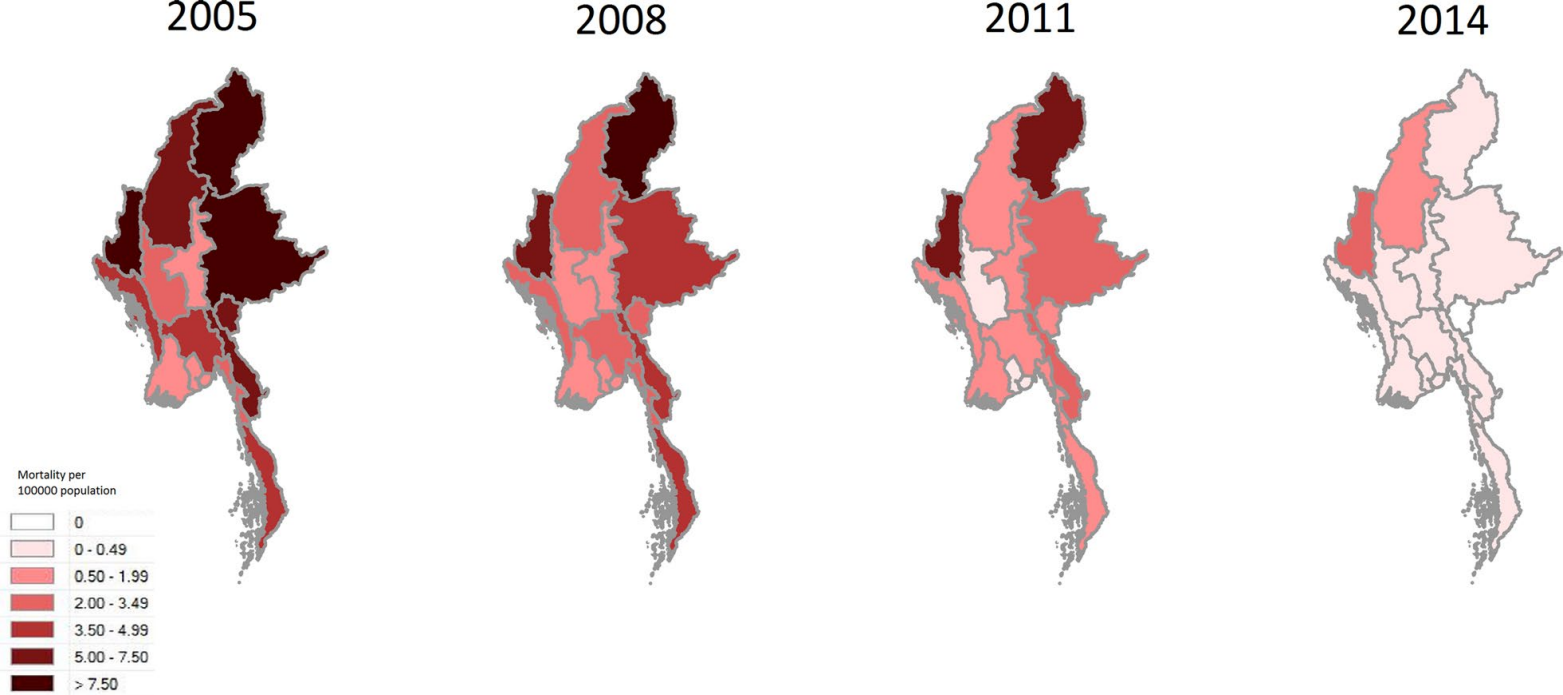
Geographical distribution of malaria deaths 2005–2014 (expressed per 100,000 population)

Over the course of the study period, the reported national malaria mortality fell from 3.79 deaths per 100,000 population, to 0.25 deaths per 100,000 population, a decline of 93.5 % (Fig. 3). The reported mortality fell in all of the states and regions of the country, ranging from a 77.3 % decline in Chin State to a 100 % decline in Kayah State (in the last 2 years of the study period there were no reported malaria deaths in Kayah State) (Fig. 4; Table 2).

**Table 2:** Malaria mortality (per 100,000 population) by region/state 2005–2014

Region/state	2005	2006	2007	2008	2009	2010	2011	2012	2013	2014	% decline^a^
Ayeyarwaddy	1.88	1.78	1.17	0.90	0.81	0.97	0.74	0.64	0.21	0.16	91.5
Bago	3.62	3.35	1.81	2.74	1.92	0.96	0.87	0.61	0.27	0.12	96.6
Chin	12.44	9.31	4.94	6.70	5.11	5.67	7.35	1.88	0.82	2.82	77.3
Kachin	19.17	21.02	12.23	8.64	15.98	7.68	5.06	3.81	2.58	0.34	98.2
Kayah	5.09	8.76	2.99	2.99	5.92	1.86	1.80	0.75	0.00	0.00	100.0
Kayin	5.32	7.09	2.11	3.55	2.16	3.65	2.30	1.44	2.18	0.48	91.0
Magway	2.16	2.28	1.70	1.66	1.02	0.54	0.24	0.15	0.22	0.07	96.6
Mandalay	1.60	1.26	0.99	1.09	1.12	0.95	0.56	0.09	0.14	0.21	87.1
Mon	3.10	2.23	2.31	2.29	1.97	1.86	1.23	0.56	0.41	0.05	98.5
Nay Pyi Taw^b^	n/a	n/a	n/a	n/a	n/a	n/a	n/a	0.10	0.31	0.00	n/a
Rakhine	4.48	5.28	2.54	2.61	2.29	1.47	1.15	0.61	0.34	0.15	96.6
Sagaing	5.38	4.86	2.65	3.39	3.71	2.42	1.68	1.56	1.06	0.82	84.7
Shan	8.17	7.08	4.03	4.05	3.01	3.45	2.25	1.14	0.88	0.23	97.2
Tanintharyi	3.96	7.57	4.94	4.80	3.28	3.60	1.61	1.17	0.44	0.14	96.4
Yangon	0.60	1.28	0.70	0.52	0.46	0.36	0.08	0.00	0.02	0.02	97.4
*National*	*3.79*	*3.86*	*2.27*	*2.37*	*2.24*	*1.73*	*1.17*	*0.73*	*0.50*	*0.25*	*93.5*

^a^Between 2005 and 2014

^b^Nay Pyi Taw was formed as an administrative region in 2010; until 2012 data were included in the Mandalay region

### Hospital admissions

**Fig. 5:**
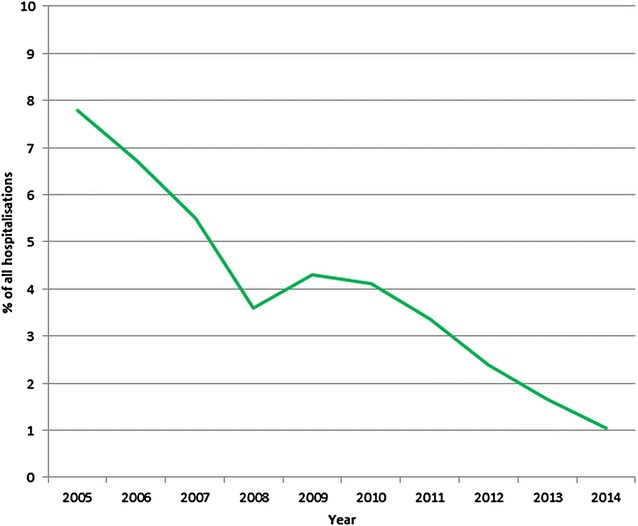
Percentage of all hospital admissions due to malaria 2005–2014

In 2005, 7.8 % of all hospital admissions in Myanmar were due to malaria. This declined by 87 % to 1.0 % of all hospital admissions in 2014 (Fig. 5). These data were not broken down by state and region.

## Discussion

There has been a marked recent decline in reported malaria incidence and mortality in Myanmar’s public health system. These national data, collected during a period of increased political and financial commitment to the rapid escalation of disease control programmes, echo the findings from smaller studies performed in different parts of the country during the same period [9, 20–23].

One of the key interventions in Myanmar has been the training and deployment of over 40,000 community health workers who complement the care provided by health care workers in rural locations which bear the greatest burden of disease [1, 15]. Although the programme has not been without its challenges [10], it is relatively inexpensive to implement [18] and allows a socio-economically disadvantaged population improved access to early and reliable diagnosis and treatment [24–26]. While the primary role of these community health workers has been the care of patients with symptoms of malaria, they also potentially have a role to play in the implementation of other malaria control activities such as the distribution of insecticide-treated nets (ITNs) and the coordination of indoor residual spraying (IRS) [14]. They might also contribute to the triage and management of other diseases, which could defray the costs of the programme [15, 27].

Myanmar commenced an ITN distribution programme in 2001. Presently there is coverage of over 60 % of the country’s at-risk population [1] and there are plans to improve this coverage to over 80 % of the at-risk population [15]. While again this programme has its challenges [12], it is likely to have significantly contributed to the decline in cases in the country over the course of the study period [28, 29]. An advantage of the ITN programme is its ability to access less developed and remote locations more effectively than other interventions [30]. Since 2010, there has been mass distribution of long-lasting insecticide-treated nets which has focussed particularly on highly mobile migrant workers, a population that is more likely to be non-immune and vulnerable to malaria than local residents [12]. Although there is DDT and pyrethroid resistance in Myanmar [1], targeted use of IRS may also be appropriate in high-transmission settings [14].

ACT has transformed the treatment of malaria, contributing to the significant decline seen in malaria incidence globally [29, 31] and it has almost certainly had a major positive impact in Myanmar. ACT has been recommended as the first-line treatment of malaria in the national malaria treatment policy since 2002 and this study period captures the rapid upscaling of programmes to improve access to ACT in both the public and private health sector. However, the recent rise of artemisinin resistance is perhaps the greatest current threat to this progress [5, 6]. Over a quarter of patients in southern Myanmar had a measurable parasitaemia 72 h after initiating artesunate therapy [32]. While on the Thai–Myanmar border, there has been a decline in PCR-adjusted cure rates with the standard regimen of artesunate-mefloquine (MAS3) from 100 % in 2003 to 81.1 % in 2013 [23]. In 2009 the Myanmar Malaria Technical and Strategy Group recommended that artemether–lumefantrine should be used as first-line therapy, however it can be anticipated that resistance will evolve to this combination in time as well [33, 34]. Dihydroartemisinin–piperaquine has been shown to be effective in Myanmar [35], but resistance has developed in other areas of Southeast Asia [36, 37] and would also be expected in Myanmar if rolled out on a larger scale [33]. Continued monitoring of the therapeutic efficacy of first- and second-line medicines with timely change of anti-malarial treatment policy is therefore essential. Future approaches may require additional novel strategies, including the use of longer ACT courses [38], sequential ACT courses [39] or the use of triple ACT (TACT), which combines partner drugs with different resistance mechanisms [39]. Mass drug administration has also been proposed [39, 40]. In the meantime it will be essential to ensure that ACT courses are completed [39], that primaquine is routinely used to sterilise gametocytes [36] and that these pharmacological approaches are complemented by more aggressive use of vector avoidance and control measures [29, 41].

It is interesting to note that four of the areas most affected by artemisinin resistance in the north and east of the country (namely Kachin State, Shan State, Kayah State, and Mon State) were the four regions with the greatest falls in mortality over the course of the study. A fifth state in the east of the country affected by artemisinin resistance, Kayin State, had a fall in mortality that was lower than the national decline, but which was still an impressive 91 %. This does not diminish the clinical significance of the artemisinin resistance; it rather reflects an awareness of the issue and an extensive investment in malaria control programmes in these regions by the national government, donors, NGOs, and partners.

However, while the evolution of artemisinin resistance has captured much of the world’s attention recently, other factors contribute to the persistence of the disease and malaria related deaths. Chin State in the northwest of the country was the region with the highest disease incidence in 2014, the highest mortality in 2014 and the lowest fall in mortality over the course of the study. This is despite the state having relatively low rates of artemisinin resistance compared to other regions in Myanmar [6]. The ongoing malaria transmission and relatively high malaria mortality in Chin State may be related less to artemisinin resistance than the fact that it is the poorest state in the country, with a dispersed population in a very mountainous region with few transportation links; all of which hamper the upscaling of effective public health interventions [42]. The Ayeyarwaddy Region had the smallest decline in malaria incidence over the course of the study. In May 2008, this region bore the greatest brunt of Cyclone Nargis, the largest natural disaster in Myanmar’s recorded history. The resulting damage to infrastructure and health systems may have contributed to the rise to pre-2005 incidence levels in the ensuing years, before the decline in incidence resumed in 2012.

The importance of factors beyond targeted malaria control programmes is underlined by the fact that the decline in malaria incidence in Myanmar began in the early 1990s, well before these malaria specific programmes were introduced. Greater government health spending, resulting in more health facilities and health care workers, has improved access to care [15]. Changing population demographics may also have contributed to the decline as there has been a significant increase in the number of people living in urban environments where malaria incidence is lower [43]. Meanwhile, the recent rate of deforestation in Myanmar has been amongst the highest in the region [44] and this is likely to have had an effect on vector populations [45].

However, the study has significant limitations and the data should be viewed with some caution. Primary health workers with little training in data management and limited access to electronic databases collected the majority of the data. It is therefore likely to be imperfect. In some locations, particularly early in the study period, access to reliable diagnostic testing was sub-optimal. It was only from 2012 that only laboratory-confirmed cases were recorded, although it should be noted that if only this 2012–2014 period is examined, the reported national malaria incidence declined by 63.1 %, and the reported national malaria-related mortality fell by 65.8 %. HMIS data include only malaria cases that are managed in the public health system and as the majority of patients with malaria in Myanmar receive their care in the private health system, in which there is no formal data collecting system, it is almost certain that the absolute incidence and mortality are higher than is reported here [15]. Determination of the population in each state and region was also problematic. The last national census completed prior to the study period was in 1983 and during the study period the population was only estimated in each region of the country, even at an official level [19]. Indeed, the 2014 census led the national population to be revised downwards from the government’s estimated figure of 60.98 million in 2012 to 51.49 million [46]. This suggests again that the incidence data here are likely to be an underestimate. However, the fact that the population figures were determined in the same manner over the entire course of the study, which was the national standard at the time, means that the temporospatial trends in the incidence data still offer valuable insights into the progress that has been made and the obstacles that remain.

There are other caveats. The HMIS data do not record the *Plasmodium* species causing malaria, which is important, as there are different challenges in eliminating *P. falciparum* and *Plasmodium vivax*. There was no formal quality assurance programme in place to confirm the reliability of the HMIS data, although this is planned in the future. Finally, while the fall in disease incidence and mortality coincided with the expansion of the aforementioned malaria-specific programmes, it was not possible to link this central HMIS data with local data on ACT prescription, ITN use and health-seeking behaviour. It is therefore not possible to state conclusively that these malaria programmes are responsible for the progress seen, although given their efficacy in other parts of the world, this inference may not be unreasonable [28, 29, 31].

While these data are positive, major challenges remain and there are many historical precedents for resurgence of malaria [47, 48]. It would be potentially catastrophic if artemisinin resistance travels from Southeast Asia to Africa, where there is a far greater burden of disease, and in many cases even more fragile public health infrastructure [1, 5]. For continued progress on a national level, it will be essential for there to be ongoing coordination and cooperation between the public sector, private sector and affected communities, particularly mobile populations [4]. At a regional level there needs to be expanded collaboration, technical support and information sharing [15].

## Conclusion

The data presented here are relatively basic; to guide policy more reliably in the future, it will be important to collect and analyse more detailed data prospectively and to link these data with the delivery of malaria control measures, the movement and health-seeking behaviour of affected populations and indices of drug resistance. It will be essential to have robust quality assurance mechanisms to ensure that the collected data are complete, timely and accurate. However, despite these issues, this study shows the enormous progress that has been made in a country recovering from over half a century of conflict. With continued political and financial commitment and health system strengthening, the goal of eliminating malaria from the Greater Mekong Region by 2030 may not be an impossible one.

## Abbreviations

ACT: artemisinin-based combination therapy
HMIS: Health Management Information System
HREC: Human Research Ethics Committee
IRS: indoor residual spraying
ITN: insecticide treated net
MAS3: 3-day regimen of mefloquine and artesunate
TACT: triple artemisinin-based combination therapy
